# Implementation of an integrated primary care prevention and management program for chronic low back pain (LBP): patient-reported outcomes and predictors of pain interference after six months

**DOI:** 10.1186/s12913-024-11031-x

**Published:** 2024-05-09

**Authors:** Sara Ahmed, Regina Visca, Amede Gogovor, Owis Eilayyan, Roderick Finlayson, Marie-France Valois, Mark A. Ware, on behalf of the McGill Réseau Universitaire Intégré de Santé et Services Sociaux (RUISSS) Center of Expertise in Chronic Pain Low Back Pain Network

**Affiliations:** 1 Faculty of Medicine, School of Physical & Occupational Therapy, McGill University, 3654 Prom Sir-William-Osler, Montreal, QC H3G 1Y5 Canada; 2 Faculty of Medicine, Family Medicine, McGill University, 5858, Chemin de La Côte-Des-Neiges 3 Floor, Montreal, QC H3S 1Z1 Canada; 3 Faculty of Medicine, Family Medicine and Emergency Medicine, Université Laval, Montreal, QC Canada; 4 Centre for Interdisciplinary Research in Rehabilitation of Greater Montreal (CRIR), Lethbridge Layton Mackay Rehabilitation, CIUSSS West-Central Montreal, Montreal, QC Canada; 5 Research Institute of the McGill University Health Center, Clinical Epidemiology, Montreal, QC Canada; 6 Centre of Expertise in Chronic Pain of the Réseau Universitaire Intégré de Santé Et Services Sociaux McGill, 1650 Cedar Ave., Montreal, QC H3G 1A4 Canada; 7 Alan Edwards Pain Management Unit of the McGill University Health Centre, 1650 Cedar Avenue, Montreal, QC H3G 1A4 Canada; 8 Department of Medicine, McGill University, Montreal, QC Canada; 9 Faculty of Applied Medical Sciences, Physical Therapy Department, Al-Ahliyya Amman University, Amman, Jordan

**Keywords:** Primary care, Low back pain, Person-centred care, Integrated care, Chronic pain, Sub-acute pain

## Abstract

**Background:**

Integrated primary care programs for patients living with chronic pain which are accessible, interdisciplinary, and patient-centered are needed for preventing chronicity and improving outcomes. Evaluation of the implementation and impact of such programs supports further development of primary care chronic pain management. This study examined patient-reported outcomes among individuals with low back pain (LBP) receiving care in a novel interdisciplinary primary care program.

**Methods:**

Patients were referred by primary care physicians in four regions of Quebec, Canada, and eligible patients received an evidence-based interdisciplinary pain management program over a six-month period. Patients were screened for risk of chronicity. Patient-reported outcome measures of pain interference and intensity, physical function, depression, and anxiety were evaluated at regular intervals over the six-month follow-up. A multilevel regression analysis was performed to evaluate the association between patient characteristics at baseline, including risk of chronicity, and change in pain outcomes.

**Results:**

Four hundred and sixty-four individuals (mean age 55.4y, 63% female) completed the program. The majority (≥ 60%) experienced a clinically meaningful improvement in pain intensity and interference at six months. Patients with moderate (71%) or high risk (81%) of chronicity showed greater improvement in pain interference than those with low risk (51%). Significant predictors of improvement in pain interference included a higher risk of chronicity, younger age, female sex, and lower baseline disability.

**Conclusion:**

The outcomes of this novel LBP program will inform wider implementation considerations by identifying key components for further effectiveness, sustainability, and scale-up of the program.

## Introduction

Chronic pain is a significant problem in the population, and prevalence increases with age [1]. In 2010, the Chronic Pain Association of Canada reported that “the annual cost of chronic pain, including medical expenses, lost income, and lost productivity, is estimated to exceed $10 billion” [2]. Over one-third of those suffering from chronic pain (CP) experienced low back pain (LBP) [3]. An international survey found that back pain is the most frequently occurring type of pain, particularly among young and middle-aged people [4]. According to the World Health Organisation (WHO), there is a 19% annual incidence of new cases of acute LBP in adults [1], of which 40% are persistent. Evidence suggests that early intervention for individuals at medium and high risk of chronicity can significantly reduce disability at 3 months post-intervention [5, 6].

To address the growing burden of LBP and ensure optimal management of patients, integrated primary care networks play an important role in delivering evidence-based LBP care [7]. Integrated care has been defined as “care that is coordinated across professionals, facilities, and support systems; continuous over time and between visits; and tailored to personal and family needs, values, and preferences (). It encompasses a range of possible methods and models of care aimed at facilitating improved patient experiences through enhanced coordination and continuity of care (. The AHRQ operationalized this in primary care as “a primary care team and another team or organization or service external to primary care work together with patients and families “using a systematic and cost-effective approach” to provide person- and family-centered care for a defined population.

Given the variation in the presentation of physical and psychosocial factors among individuals with chronic pain, a tailored approach that matches interdisciplinary care to individuals’ needs is necessary [8]. Evidence-based clinical guidelines, such as the McGill Spine Algorithm and others [9] as well as published data on risk factors of chronicity [10] support the development of an interdisciplinary, patient-centered and integrated model of care at the primary level for patients with chronic pain.

Self-management, physical and psychological therapies, and some forms of complementary medicine are recommended as first lines of treatment, with less emphasis on pharmacological and surgical treatments [7, 11]. Guidelines encourage active treatments that address psychosocial factors and focus on functional improvement ([1, 12]. Early interdisciplinary diagnosis and treatment of chronic pain have been associated with decreased risk of chronicity, improved return to work, and quality of life outcomes ([1, 13]. Guideline recommendations also include strengthening consultation between professionals and improving access to and coordination of care at the primary, secondary and tertiary levels [14–16].

Despite this potential, many gaps exist in the optimum management of LBP. Challenges include lack of awareness of guidelines and lack of access to interdisciplinary teams. There is fragmentation, or lack of continuous, comprehensive, and coordinated care, between different levels of the health system i.e. primary, secondary, and tertiary levels of care [1, 17–19]. As a result, patients living with LBP may not receive services to address their needs in a timely manner. Lack of access to LBP care that is concordant with best practice guidelines increases risk of chronicity and long-term disability, significantly reducing the chances and costs of recovery.

To address these challenges, four health regions in Quebec, Canada developed and implemented an interdisciplinary primary care program, based on evidence-based guidelines, targeting individuals with sub-acute and chronic nonspecific LBP. Here we report the initial evaluation of the planned outcomes of this pilot program.

## Methods

### Design

This was an observational study using quantitative and qualitative approaches [20, 21] to evaluate the program over six months. Data collection was managed using the Research Electronic Data Capture [22] (REDCap). The details for the co-creation of the program and implementation evaluation, and qualitative methods and results have been presented elsewhere [23].

#### Low back pain model of care

An assessment of pain management needs was conducted by the provincial Center of Expertise in Chronic Pain by establishing the governance committees that oversaw the development and implementation of the LBP program. The program was co-developed using an integrated knowledge translation approach with two patient partners and representatives from clinical care, education, and research and the ministry of health and social services [24]. Based on these consultations an interdisciplinary program was designed to offer patients with chronic LBP access to a team including a nurse, a physiotherapist, a psychologist, and a physician, which incorporated evidence-based back pain management practices and self-management support [25]. The interdisciplinary team members were present at the first visit, at the same time, with the patient and each completed their assessment. This allowed the team to avoid asking the patient the same questions, and each team member was able to learn about the patients LBP by being present during the assessments of all team members. The program further defined services across the care continuum with referral criteria to facilitate the transition to secondary and tertiary care services when required [26].

Primary care physicians learned about the program from regional nurses that visited the primary care provider clinics to share information about the LBP program, and provided a paper copy of the decision algorithm for the clinical trajectory for referral to the program (Fig. 2), and referral form. Primary care providers faxed referral to the LBP program, and the program nurse triaged patients for eligibility for the program. If a patient was not eligible for the program, the nurse contacted the primary care provider to inform them that the patient did not meet the inclusion criteria with an explanation.

**Fig. 1:**
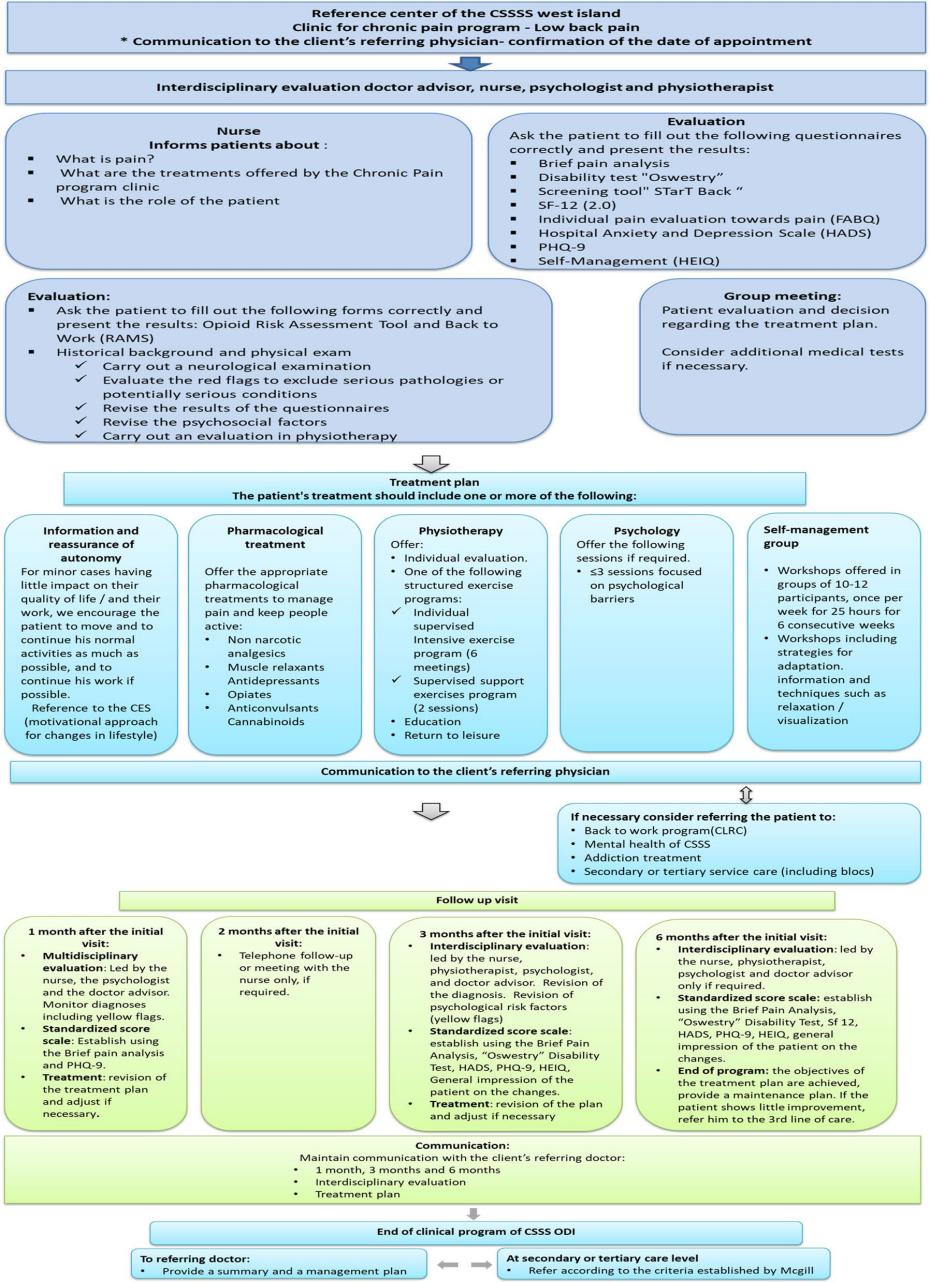
Treatment of non-specific subacute low back pain. McGill RUISSS Center of expertise clinical chronic pain program

**Fig. 2:**
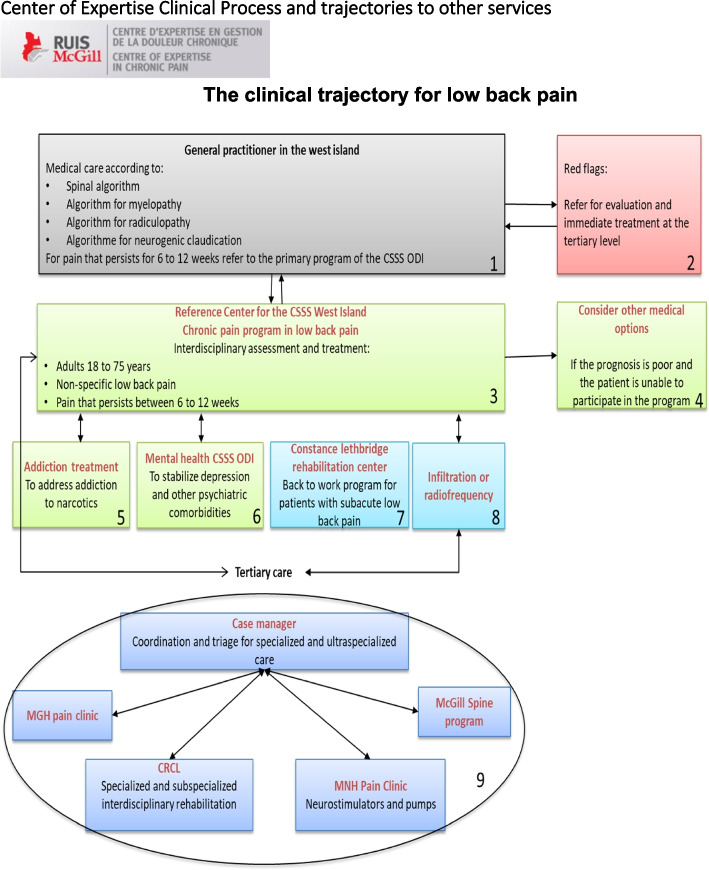
McGill RUISSS. Center of expertise clinical process and trajectories to other services

Valid and standardized assessment tools were used to identify patients’ treatment needs [24, 27–30]. Adopting an interdisciplinary and patient-centered approach, the care team completed the initial baseline patient evaluation together, and developed a treatment plan which was discussed and agreed with the patient (Fig. 1). Interdisciplinary follow-up was planned at 1.5, 3, and 6 months after the initial visit, with additional telephone follow-up by the nurse at 4.5 months to ask the patient if there was any deterioration in their condition to plan for a visit earlier than 6 months if needed. Where needed, the team coordinated follow-up with additional professionals (e.g., occupational therapist, social worker, etc.) and referrals to other specialized services. As defined in the algorithm (Fig. 2), corridors of service were established between primary, secondary and tertiary medical and rehabilitation services to address outcomes that were not improving in the program, including depression, addiction and issues related to return to work.

Core elements for the treatment plan were defined for each discipline, focusing on empowering patients to develop the skills needed to manage symptoms and improve function. In addition, the interdisciplinary teams were trained to apply the 5A strategy to help individuals set realistic goals and define an action plan to develop the skills needed to achieve these goals. The 5As [31] focus on clinician completion of five specific tasks (Ask, Advise, Assess, Assist, Arrange), each supported by evidence [32, 33], which are necessary to effectively counsel patients about health habits and skills needed to better manage their chronic condition.

#### Study sites and participants

The program was implemented in four local health regions (i.e., Centre de Santé et de Services Sociaux). In each health region, primary care physicians were informed how to refer eligible patients by regional nurses. Inclusion criteria (which evolved during pilot phase implementation) were age 18–75; diagnosis of sub-acute LBP; eventually expanded to also include chronic LBP (≤ 1 year duration) based on primary care phsyciains request given some patients seek care beyond 3 months, however, 1 year was selected to ensure patients were in earlier phases of the chronicity; and proficiency in French or English. Limiting age to 75 years old was based on older adults being more likely to develop certain LBP pathologies (e.g., osteoporotic vertebral fractures), and various age-related physical, psychological, and mental changes (e.g.,age-related changes in central pain processing). Exclusion criteria included: ongoing coverage by workplace insurance (e.g. Commission de la santé et de la sécurité du travail CSST); the presence of ‘red flags’ including recent weight loss, fever, significant neurological damage; or the presence of symptoms and signs suggesting emergency conditions such as cauda equina syndrome.

### Measures

Patients completed a set of validated Patient Reported Outcome Measures (PROMs) and questionnaires electronically in English or French in the waiting room at baseline, three and six months. The PROMs were selected by the evaluation committee (part of the governance described above) with support of the research team to capture outcomes important for interdisciplinary treatment planning and patient partners. The questionnaire took 35–45 min to complete. The first section evaluated the impact of pain, physical and mental health, functional status, quality of life, current roles, and quality of care and follow-up. These measures included the Oswestry Disability Index (ODI) designed to assess limitations of various activities of daily living [27], the Brief Pain Inventory (BPI) that provides a valid means of measuring pain intensity and the extent to which pain interferes in the lives of the pain sufferers [28]; the Fear Avoidance Beliefs Questionnaire (FABQ) that assesses patients’ beliefs about how physical activity and work affected their low back pain [34]; the Hospital Anxiety and Depression Scale (HADS) used for detecting states of depression and anxiety in the setting of a hospital medical outpatient clinic and the severity of the emotional disorder; the Patient Health Questionnaire (PHQ-9) that assesses for the presence of a depressive disorder [24]; the RAND-12 designed to measure general health status that includes 8 concepts (physical functioning, role functioning physical, bodily pain, general health, vitality, social functioning, role functioning emotional, and mental health) and the results can be expressed in terms of two meta-scores: the Physical Component Summary (PCS) and the Mental Component Summary (MCS); and the Patient Global Impression of Change (PGIC) [29]. The pain self-efficacy scale was administered to assess individuals’ confidence in carrying out activities despite their pain [30]. The Patient Assessment of Chronic Illness Care (PACIC) questionnaire asked about specific actions or qualities of care congruent with the Chronic Care Model (CCM) that patients report they have experienced in the delivery system.

The last part of the questionnaire collected socio-demographic information and information about the individuals’ primary care doctor and clinic. For individuals who did not return to the program after the initial visit, an exit questionnaire was administered to ask patients why they did not return, whether they are receiving services elsewhere and whether their insurance coverage has changed.

#### Statistical analyses

Changes in PROMs were evaluated by calculating the percentage of participants that experienced an improvement greater than the minimally clinically important difference (MCID). Based on published estimates, MCID were calculated as 0.5 SD change for all measures except for depression and anxiety for which the MCID is 2.1 and 1.9, respectively [35, 36]. Participants were classified on each PROM as no change, or deterioration for each PROM. Changes in PROMs were evaluated between baseline (initial visit) to 3 months and between baseline to 6 months. Subgroup analyses were conducted to compare the proportion that experienced a MCID across three sub-groups of the Keele STarT-Back (low, medium, and high risk of chronicity) at baseline.

We conducted hierarchical multivariable regression to evaluate the relationship between patient (demographic variables in Table 3) and process covariates (clinical/program variables in Table 4) and the change in pain interference as the outcome. The difference was calculated as difference in pain interreference at 6 months minus the baseline value. Therefore, a negative value indicated a better/improvement (i.e. 6-month pain was smaller than baseline pain). Similarly, a positive value indicated worse/deterioration since 6-month pain was higher than baseline pain. We first estimated univariate models, and all significant covariates (95% confidence interval does not include the null value) in the unadjusted models were included in the multivariate model. Exceptions were covariates that had a high correlation with other covariates or had missing data that exceeded 10%, which were not included in the model. The exception was sex, which we felt was important to include in the multivariate model, even if it was not significant in the univariate model given the importance of sex differences in response to interventions [37]. The analyses were performed using SAS version 9.4. (SAS Institute, Cary, NC, USA).

### Ethical considerations

All procedures were conducted in accordance with the ‘Declaration of Helsinki’ Ethics approval for this study was obtained from the McGill University Health Center IRB (#MP-CUSM-12–220 GEN/2013–999). Written informed consent was obtained from all the participants.

## Results

### Characteristics of the participants

Between May 2012 and March 2017, 24 primary care physicians referred 1545 individuals to the four programs. Among these, 321 (21%) individuals could not be reached after referral, and 752 (49%) were accepted into the program of which 689 (92%) consented to use their data for evaluation purposes. Reasons for not being accepted into the program were available for 135 individuals (29%) and were mainly related to not meeting the inclusion criteria for age (*n* = 47), the duration of pain (*n* = 71), or pain resolving (*n* = 41), or having a red flag (*n* = 46). Among the 689 who consented, a total of 225 (33%) dropped out, and 464 (67%) completed questionnaires at 3 months. The average age of the 464 participants was 55y (SD = 15) and 63% were female. Twenty-four percent had a mild risk of chronicity, 33% a moderate risk, and 27% a severe risk.

Among those who completed the program, they had an average of 1.5 visits with the physician, 2.3 visits with the psychologist, 3.4 visits with the nurse, and 5.3 visits with the physiotherapist. Between the first visit and 6 months, most individuals had at least one visit with all interdisciplinary team members (56.69%). Those that saw all members except for the physician on the team represented 24.84%, and 8.92% saw all members except for the psychologist. The remainder of patients had at least one visit with the physiotherapist and psychologist (2.55%), or nurse and psychologist (0.6%), the nurse and physiotherapist (3.18%), all members except the nurse (1.91%), all members except for the physiotherapist (0.64%), and physician and physiotherapist (0.32%). The remainder only had a visit with the physiotherapist (0.96%).

Those who did not complete the program (*n* = 225) had slightly higher levels of pain interference, anxiety, and depression at baseline as compared to those that remained in the program (*n* = 464) (Table 1). Among those who dropped out compared to those who remained in the program, there was a slightly higher proportion in the high-risk chronicity group. Reasons for not continuing in the program were available for 110 individuals (49%) and were variable (Table 2).

**Table 1:** Baseline characteristics of participants at baseline

Variable (Range)	Stayed in *N* = 464; Mean (sd)	Dropped-out *N* = 225; Mean (sd)	*p*-value^§^
Age	55.4 (14.8)	49.2 (16.2)	< 0.0001
Gender (% Female)	294 (63%)	132 (59%)	0.2208
Pain Severity (0–10)	4.7 (1.9)	5.2 (2.0)	0.0011
Pain Interferences (0–10)	4.6 (2.4)	5.3 (2.4)	0.0017
HADS-Depression	6.2 (4.1)	7.3 (4.2)	0.0027
HADS-Anxiety	8.7 (3.8)	9.3 (4.0)	0.1086
Oswestry	32.8 (15.5)	36.4 (15.7)	0.0054
Physical Health (0–100)	35.6 (10.1)	34.7 (9.5)	0.3527
Mental Health (0–100)	47.6 (11.2)	44.1 (10.8)	0.0008
Keels Start Back (0–9)	4.9 (2.3)	5.5 (2.3)	0.0037
Mild	112 (24%)	42 (19%)	
Moderate	155 (33%)	68 (30%)	
Severe	127 (27%)	81 (36%)	0.1004

^§^*p*-value from t-test for continuous variables and chi-square for categorical variables

**Table 2:** Reason for refusing to remain in the program

Reason	Frequency
No interest in the program	18
No reason mentioned	22
Needs do not correspond to program	6
No more pain	5
Not available due to changes in circumstances (e.g. work schedule, caring for family member, moved)	24
File closed by the team (eligibility changed)	4
Other medical incident (e.g.fall, surgery, stroke)	10
Follows the recommendation of physician, physician says program not adequate for her	1
Could not reach	15
Language barrier	2
Too far	2

### Impact on physical, mental and social health

**Fig. 3:**
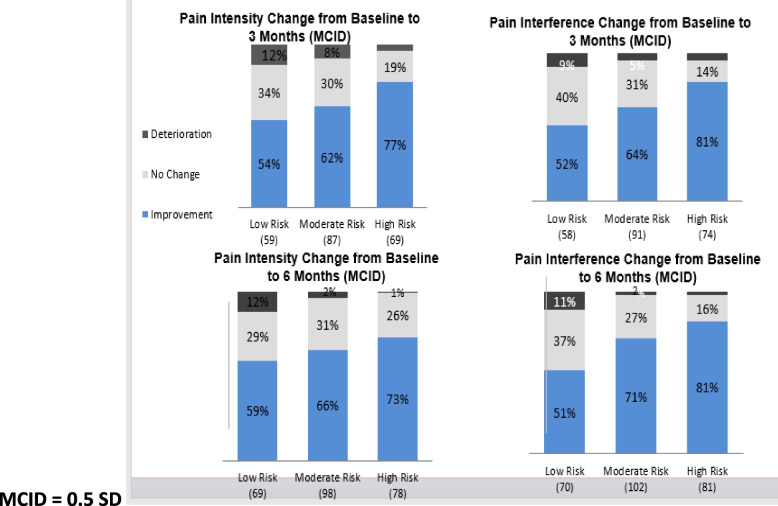
Minimal Clinical Important Difference (MCID) for pain intensity and pain interference. MCID = 0.5 SD

**Fig. 4:**
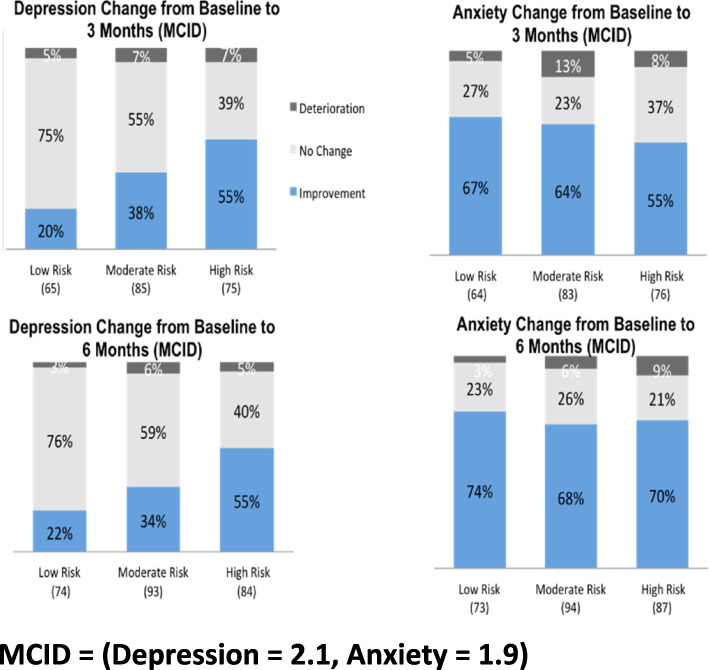
Minimal Clinical Important Difference (MCID) for depression and anxiety. MCID = (Depression = 2.1, Anxiety = 1.9)

**Fig. 5:**
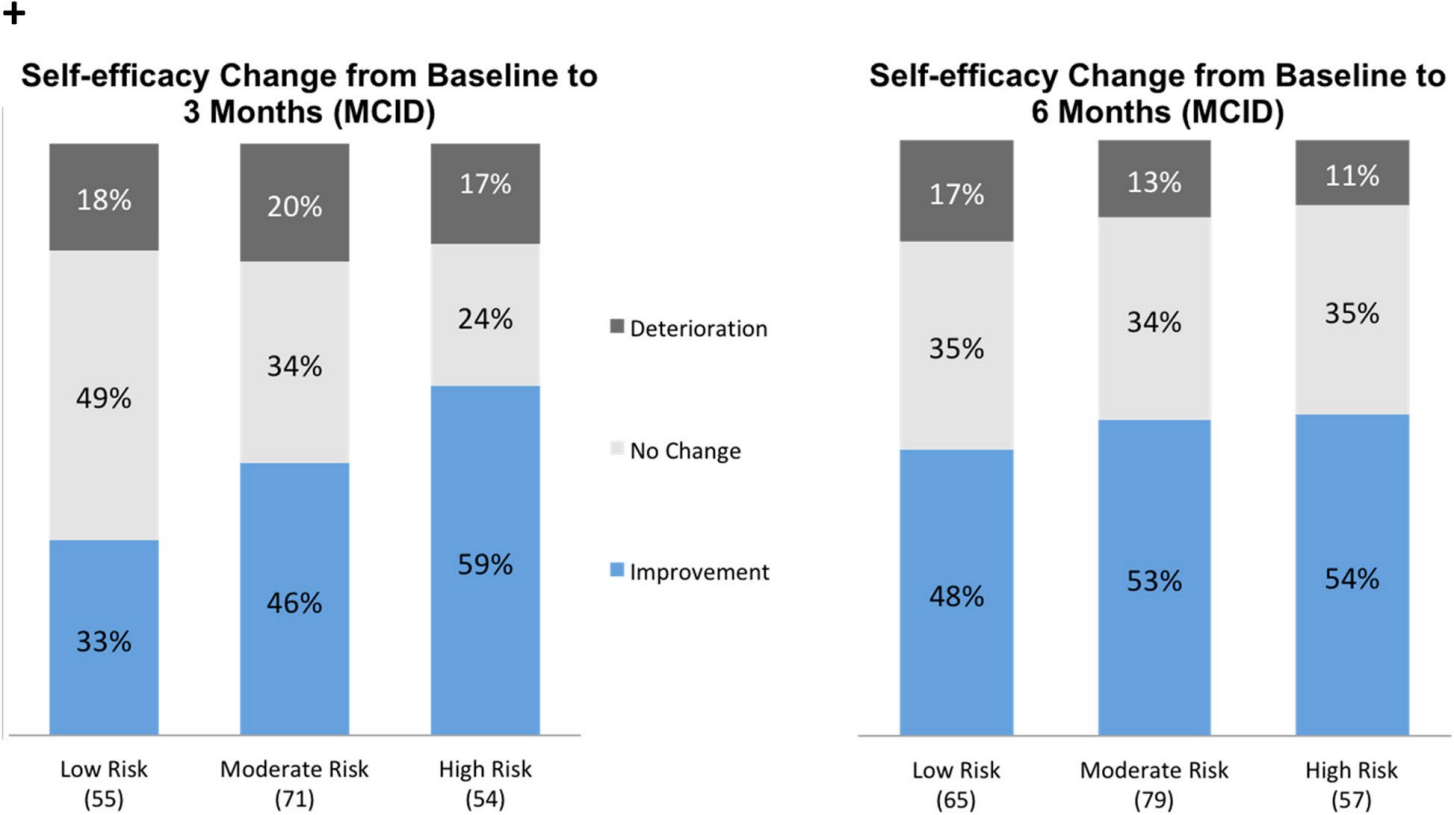
Minimal Clinical Important Difference (MCID) for self-efficacy

One hundred forty-eight (68%) and 163 (67%) experienced a clinically meaningful improvement in pain intensity at three and six months, respectively. Similarly, 139 (62%) and 174 (69%) experienced a clinically meaningful improvement in pain interference at three and six months, respectively. A greater proportion of those in the moderate (71%) or high-risk (81%) of chronicity group showed an improvement in pain interference than in the low-risk group (51%) at six months (Fig. 3). The pattern between risk of chronicity groups was the same for pain intensity. For depression, the proportion that had a clinically meaningful improvement, at six months, in the moderate (34%) and high-risk (55%) groups was higher compared to the lower-risk group (22%)( Fig. 4). In contrast, a greater proportion of the low-risk group (74%) experienced a clinically meaningful improvement in anxiety at 6 months compared to the moderate (68%) and high-risk (70%) groups (Fig. 4). A larger proportion of individuals in the moderate (46%) and high-risk (59%) groups also experienced a clinically meaningful improvement in self-efficacy at 3 months compared to the low-risk group (33%) (Fig. 5). This pattern remained up to 6 months, however, the differences were not as large (low risk = 48%, moderate risk = 53%, high risk = 54%) (Fig. 5).

Tables 3 and 4 present the univariate relationship between the measures and covariates and pain interference changes over six months. The multivariate model revealed significant predictors of change in pain interference between baseline and six months (Table 5). Significant predictors included prognostic risk of chronicity (Start Back) (MC [CI] _high/low_ -0.6[-1.4,0.3]; MC [CI] _medium/low_ –0.4[-1.1, 0.3]), age (for an increase of 10 years, 0.2[0.0,0.4]), sex was borderline significant (95% CI is -0.99 to 0.05) and baseline disability (Oswestry Disability index- increase of 20 units (-0.5[-0.9, -0.1]). Similar to the univariate model, the number of months since implementation of the program did not emerge as a significant predictor in the multivariate model.

**Table 3:** Univariate association between demographic variables and change in pain interference between baseline and 6 months

(*n* = 314 patients)	Mean Change	95% CI	*R*^2^
Age (increase of 10 years)^a^	0.23	(0.05, 0.41)	0.022857
Age			
35	Ref^b^		0.023575
35 to 44	0.43	(-0.64, 1.50)	
45 to 54	0.75	(-0.24, 1.73)	
55 to 64^a^	1.01	(0.02, 2.00)	
> 65^a^	1.06	(0.12, 2.00)	
Male vs Female	0.00	(-0.54, 0.53)	0.000000
Marital status			
Married or Common Law	Ref		0.030690
Divorced or separated	0.68	(-0.15, 1.51)	
Never Married	0.08	(-0.75, 0.91)	
Widowed	-1.11	(-2.60, 0.39)	
Other, please specify	0.77	(-0.73, 2.26)	
Missing	0.85	(-0.12, 1.81)	
Education			
College or University	Ref		0.007779
Secondary School	-0.15	(-0.80, 0.50)	
Primary School or None	-0.70	(-2.14, 0.74)	
Other	-0.44	(-1.71, 0.83)	
Missing	0.27	(-0.58, 1.13)	
Employment			
Full time	Ref		
Part-time	-0.62	(-1.58, 0.34)	0.058075
Retired	0.41	(-0.24, 1.06)	
On disability	0.86	(-0.26, 1.99)	
Other	-0.86	(-1.73, 0.00)	
Missing	0.96	(-0.01, 1.94)	
Private Insurance			
Yes	Ref		0.007569
No	-0.40	(-1.04, 0.24)	
Missing	0.22	(-0.65, 1.08)	
Type of drug plan			
Private Plan	Ref		0.003572
Gov’t Funded Pharmacare	-0.17	(-0.88, 0.53)	
Missing	-0.30	(-0.90, 0.30)	
On social assistance			
No	Ref		0.004831
Yes	0.51	(-0.62, 1.65)	
Missing	0.36	(-0.53, 1.26)	
Canadian pension			
No	Ref		0.009422
Yes	0.48	(-0.14, 1.10)	
Missing	0.35	(-0.48, 1.17)	
Ethnicity			
Caucasian	Ref		0.012044
Other	0.38	(-0.39, 1.15)	
Missing	0.57	(-0.08, 1.23)	

^a^Significant values

^b^*Ref* Reference group

**Table 4:** Univariate association between clinical/program variables and change in pain interference between baseline and 6 months

(*n* = 314 patients)	Mean Change	95% CI	*R*^2^
Start Back			
Low	Ref^c^		0.106154
Medium^b^	-1.16	(-1.81, -0.51)	
High^b^	-1.90	(-2.59, -1.21)	
Missing^b^	-1.75	(-2.67, -0.83)	
Number of visits to			
Nurse	0.06	(-0.07, 0.20)	0.002939
MD	-0.09	(-0.25, 0.07)	0.004579
Physiotherapist	-0.06	(-0.26, 0.15)	0.001109
Psychologist	0.05	(-0.09, 0.19)	0.002016
Adherence to program: Nurse			
No (< 6 visits)	Ref		0.000003
Yes (≥ 6 visits)	-0.01	(-0.61, 0.60)	
Adherence to program: Physiotherapist			
No (< 6 visits)	Ref		0.002349
Yes (≥ 6 visits)	-0.27	(-0.94, 0.39)	
Number of months since implementation	0.00	(-0.02, 0.02)	0.000327
Within 12 months of implementation	-0.15	(-0.85, 0.54)	0.000692
^b^FABQ—Physical Activity^b^	-0.07	(-0.11, -0.02)	0.034749
^a^FABQ—Work	-0.01	(-0.03, 0.01)	0.002723
Baseline Pain Severity^b^	-0.50	(-0.63, -0.38)	0.200287
Baseline Pain Inference^b^	-0.55	(-0.64, -0.46)	0.353137
Baseline HADS – Depression, mean (SD) ^b^	-0.11	(-0.18, -0.05)	0.041907
Baseline HADS – Depression			
Minimal (≥ 0 and < 8)	Ref		0.027882
Mild (≥ 8 and < 11)	-0.52	(-1.37, 0.32)	
Moderate (≥ 11 and < 16)^b^	-1.00	(-1.83, -0.16)	
Severe (≥ 16)	-1.09	(-2.67, 0.48)	
Missing	-0.46	(-1.51, 0.59)	
Baseline HADS – Anxiety, mean (SD)^b^	-0.08	(-0.14, -0.01)	0.018080
Baseline HADS – Anxiety			
Minimal (≥ 0 and < 8)	Ref		0.032402
Mild (≥ 8 and < 11)	-0.65	(-1.34, 0.04)	
Moderate (≥ 11 and < 16)	-0.49	(-1.18, 0.21)	
Severe (≥ 16)^b^	-1.47	(-2.56, -0.37)	
Missing	-0.20	(-1.32, 0.92)	
Baseline PHQ9, mean (SD)^b^	-0.08	(-0.12, -0.03)	0.044974
Baseline PHQ9			
No depression (≥ 0 and < 5)	Ref		0.06100
Minimal (≥ 5 and < 10)^b^	-0.87	(-1.49, -0.24)	
Mild (≥ 10 and < 15)^b^	-1.26	(-2.09, -0.43)	
Moderate (≥ 15 and < 20)	-0.59	(-1.67, 0.48)	
Severe (≥ 20)^b^	-1.77	(-2.93, -0.62)	
Missing	-1.05	(-2.15, 0.05)	
Baseline ODI total, mean (SD)^b^	-0.05	(-0.07, -0.04)	0.132580
Baseline ODI total			
Minimal disability (≥ 0 and < 20)	Ref		0.102976
Moderate disability (≥ 20 and < 40)^b^	-1.17	(-1.85, -0.49)	
Severe disability (≥ 40 and < 60)	-1.79	(-2.58, -1.00)	
Crippled (≥ 60 and < 80)	-2.59	(-3.93, -1.24)	
Bed bound (≥ 80 and ≤ 100)	-3.57	(-5.74, -1.39)	
Missing	NA		
Baseline Self Efficacy^b^	0.16	(0.01, 0.30)	0.022020
Baseline RAND 12 – Physical^b^	0.07	(0.04, 0.10)	0.100486
Baseline RAND 12- Mental^b^	0.04	(0.01, 0.07)	0.040176
TCI score (change of 1 unit)	-0.85	(-1.85, 0.14)	0.010245
^b^PACIC change			
Improved	Ref		0.005465
Stable	-0.03	(-0.91, 0.85)	
Worsen	-0.35	(-0.96, 0.27)	

^a^*n* = 24

^b^Significant values

^c^*Ref* Reference group

**Table 5:** Multivariate model for mean change in pain interference between baseline and 6 months (*n* = 314)

	**Model 2** **(234 patients, *R***^**2**^** = 0.265913)**	
	**Mean Change**	**95% CI**
Age (increase of 10 years)^a^	0.18	(0.00, 0.35)
Male vs Female	-0.47	(-0.99, 0.05)
Start Back		
Low	Ref^b^	
Medium	-0.39	(-1.08, 0.30)
High	-0.56	(-1.40, 0.29)
Missing	-0.87	(-1.85, 0.10)
Number of months since implementation	0.00	(-0.02, 0.02)
Baseline Pain Severity^a^	-0.37	(-0.54, -0.19)
Baseline ODI total (increase of 20)^a^	-0.50	(-0.93, -0.07)

^a^Significant values

^b^*Ref* Reference group

## Discussion

The research evidence to date supports greater use of the biopsychosocial model for the management of individuals with LBP [38–40]. Achieving a comprehensive care model that addresses individuals’ physical, mental, and social needs requires collaborative care across health professionals. We present results from the evaluation of a novel integrated interdisciplinary program in primary care, embedded in each health region, which provided individuals with LBP with a biopsychosocial evaluation to develop a personalized approach to the complexity of their needs. Compared to previous programs for LBP, the one presented here had all team members seeing the patient on the same day and time. This strengthened the interdisciplinary communication and collaboration in developing the treatment plan for patients, and based on interviews with patients in the program published in a previous study contributed to patient satisfaction and adherence with the program.

We show that for patients who adhere to the program, there are significant improvements in pain intensity and interference as well as depression symptoms over six months. Almost 50% of those referred to the program were eligible, preventing potentially unnecessary referral to tertiary-level care. The program included personalized access to physician, nurse, physiotherapist, and psychologist; in general patients had a larger number of visits to the physiotherapist. Emphasizing physical activity and exercise was the program’s intended focus, in line with LBP clinical practice guidelines.

The majority of individuals showed clinically meaningful improvement in physical and mental health outcomes. The propostion who had a meaningful improvement was greater among those in the moderate and high-risk chronicity group compared to the low risk group as is expected given their baseline values on all measures was worse. This is similar to other studies that found that individuals with greater physical and mental health limitations benefit more from interdisciplinary care than those in the low-risk group.

We evaluated the clinical program and individual factors that are significantly associated with changes in pain interference throughout the program. We found that those in a younger age group and those with a higher risk of chronicity and disability at baseline benefited the most in terms of improvements in pain interference. These are also sub-groups of individuals most likely to experience deteriorations in function if intervention is not started early [41], increasing the risk of opioid dependency [42].

The results to date indicate that the interdisciplinary program has the potential to improve patients’ health and well-being, and their ability to engage in activities of daily living, work, and social activities. Successful sustainability and scale up of the program may also result in cost savings for patients as well as society by averting lost work days and emergency department visits. The implementation of the Chronic Pain LBP program across four different health regions provides preliminary support to evaluate the effectiveness of the program in a larger pragmatic trial that will examine outcomes beyond 6 months and costs. The implementation evaluation reported elsewhere also provides support for the acceptability and appropriateness of the program as perceived by clinicians and patients [23]. Implementation results also provided insights into how to tailor the program to individuals’ needs and risk of chronicity to increase the likely gain in outcomes relative to costs in future evaluations [43].

### Limitations

While the program’s design and evaluation provided a strong base to launch the primary care interdisciplinary program within an integrated care network, there are limitations to this work. Mainly, as this was a repeated measures study design, we did not include a control group. Some of the changes in outcomes that occurred can be expected to occur spontaneously without intervention. Further, dropouts before the end of the 6-month program occurred, mainly among those that experienced improvements early on in the program or did not want to commit the time to the program.

## Data Availability

The datasets generated and/or analyzed during the current study are not publicly available due to pending approval from the ethics committee to share the data but will be available from the corresponding author upon reasonable request.
